# Impact of oil spills on coral reefs can be reduced by bioremediation using probiotic microbiota

**DOI:** 10.1038/srep18268

**Published:** 2015-12-14

**Authors:** Henrique Fragoso ados Santos, Gustavo Adolpho Santos Duarte, Caio TavoraCoelho da Costa Rachid, Ricardo Moreira Chaloub, Emiliano Nicolas Calderon, Laura Fernandes de Barros Marangoni, Adalto Bianchini, Adriana Haddad Nudi, Flávia Lima do Carmo, Jan Dirk van Elsas, Alexandre Soares Rosado, Clovis Barreira e Castro, Raquel Silva Peixoto

**Affiliations:** 1 BIOINOVAR/LEMM – Laboratory of Molecular Microbial Ecology, Institute of Microbiology Paulo de Góes, Federal University of Rio de Janeiro (UFRJ), Rio de Janeiro, RJ, Brazil; 2 Departamento de Invertebrados, Museu Nacional, Universidade Federal do Rio de Janeiro, Rio de Janeiro, RJ, Brazil; 3 Instituto Coral Vivo, Rio de Janeiro, RJ, Brazil; 4 Department of Biochemistry, Institute of Chemistry, Federal University of Rio de Janeiro, Rio de Janeiro, RJ, Brazil; 5 Programa de Pós-Graduação em Oceanografia Biológica, Instituto de Oceanografia, Universidade Federal do Rio Grande, Rio Grande, RS, Brazil; 6 Instituto de Ciências Biológicas, Universidade Federal do Rio Grande, Rio Grande, RS, Brazil; 7 Laboratório de Estudos Marinhos e Ambientais, PUC, Rio de Janeiro, RJ, Brazil; 8 Department of Microbial Ecology, Centre for Ecological and Evolutionary Studies, University of Groningen, Groningen, The Netherlands

## Abstract

Several anthropogenic factors, including contamination by oil spills, constitute a threat to coral reef health. Current methodologies to remediate polluted marine environments are based on the use of chemical dispersants; however, these can be toxic to the coral holobiont. In this study, a probiotic bacterial consortium was produced from the coral *Mussismilia harttii* and was trained to degrade water-soluble oil fractions (WSFs). Additionally, we assessed the effect of WSFs on the health of *M. harttii* in tanks and evaluated the bacterial consortium as a bioremediation agent. The consortium was responsible for the highly efficient degradation of petroleum hydrocarbons, and it minimised the effects of WSFs on coral health, as indicated by raised photosynthetic efficiencies. Moreover, the impact of WSFs on the coral microbiome was diminished by the introduced bacterial consortium. Following introduction, the bacterial consortium thus had a dual function, i.e promoting oil WSF degradation and improving coral health with its probiotic features.

Coral reefs are amongst the most diverse and productive ecosystems on Earth, and they play a key role in the maintenance of ocean functions^1^. However, reefs are vulnerable to various human activities^2^,^3^,^4^,^5^, including contamination by oil spills. Reef protection against human impacts (e.g. overfishing, pollution and climate change) is a global challenge^6^. Approximately 8.4 million tons of petroleum products diffuse into the oceans each year^7^. Unfortunately, large fractions of this load may potentially impact coral reef ecosystems. Additionally, coral reef locations are at risk, as oil tankers may suffer accidents at such oceanic sites^8^. So far, the magnitude of the effects of oil spills on coral systems remains unknown. Moreover, current methodologies to remediate polluted marine environments are based on the use of chemical dispersants, which can be toxic to the holobiont^9^.

Corals are key eukaryotic organisms that have intricate relationships with a suite of eukaryotic and prokaryotic microorganisms, including endosymbiotic dinoflagellates (zooxanthellae), fungi, bacteria and archaea^10^,^11^,^12^,^13^,^14^. Intricate and only partially understood relationships across all members of the holobiont are key to the maintenance of coral health. It has been suggested that the coral microbiome is involved in a plethora of interactive functions^15^,^16^. In fact, microbiome studies from other hosts have provided new insights into how microorganisms control host health, either as pathogens or as symbionts^16^. The recent ‘coral probiotic hypothesis’^12^ suggests that the holobiont can adapt to stressful conditions by modulating its microbial diversity and community structure to improve coral health and resilience. However, it remains unknown to what extent the microbiome may protect corals from oil pollution. Moreover, this lack of knowledge undermines the potential use of such microorganisms for the bioremediation of contaminated coral reef areas. Can these be employed to ‘strengthen’ corals under stress such as that caused by oil spills?

Considering the need to understand the role of the coral microbiome as a modulator of coral health under oil stress, we here addressed (1) the impact of oil WSFs on coral health and (2) the potential of microbially-driven coral protection and oil bioremediation. For this study, we used a coral-isolated microbial consortium adapted to the coral/seawater habitat.

## Results

### Bacterial consortium

Partial 16S rRNA gene sequences revealed that the bacterial consortium with 10 morphotypes cultured from *M. harttii* was composed of organisms affiliated with *Bacillus rigui* (strain I1, 99% homology), *Acinetobacter calcoaceticus* (strain I2, 99%), *B. catenulatus/indicus/cibi* (strain I3, 96%), *B. aryabhattai* (strain I4, 99%), *Paracoccus homiensis* (strain I5, 99%), *P. kamogawaensis* (strain I6, 99%), *P. marcusii* (strain I7, 99%), *Psychrobacter sp*. (strain I8, 99%), *Vibrio alginolyticus* (strain I9, 99%) and *Pseudomonas stutzeri* (strain I10, 99%).

### Impact of oil WSFs and potential of the bacterial consortium to improve coral health/survival

To evaluate the impact of oil WSFs on coral health and to study the potential modulation of coral health by the bacterial consortium, different parameters were analysed in microcosm systems as described below.

### Efficiency of degradation of oil WSFs by the bacterial consortium

**Figure 1:**
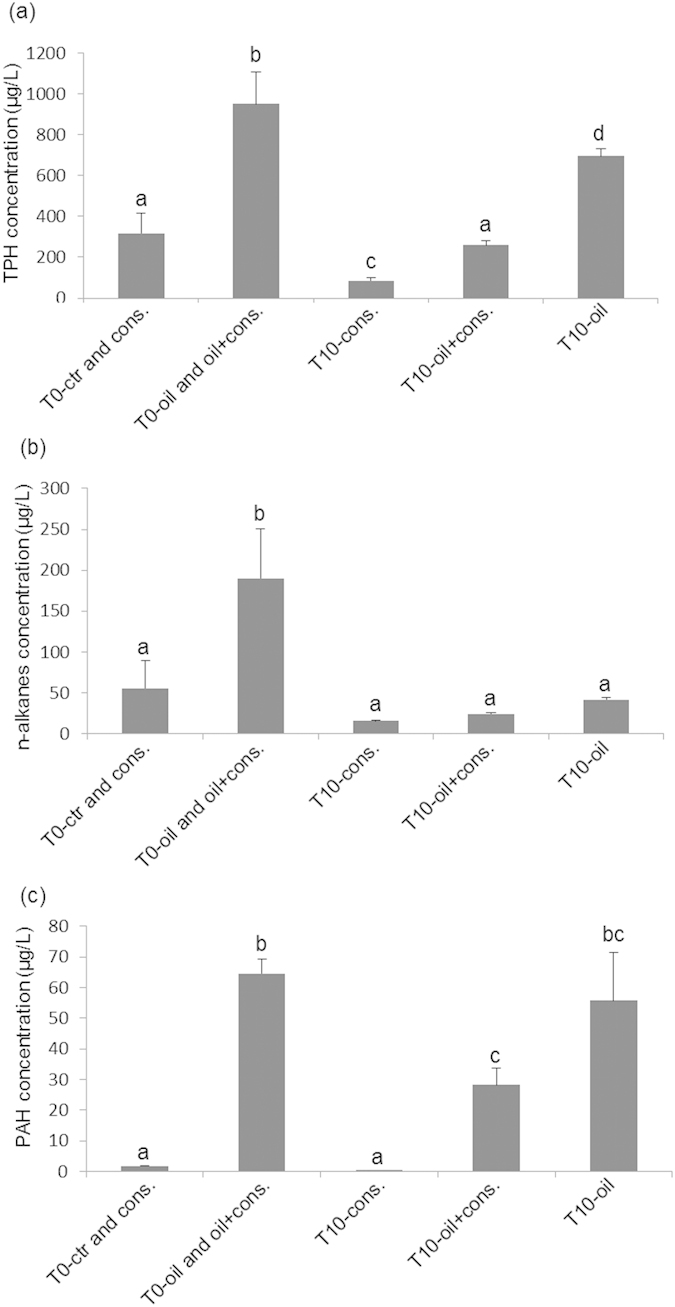
Concentrations of hydrocarbons during experimental sampling. Concentration of total petroleum hydrocarbons (TPH) (**a**). concentration of n-alkanes hydrocarbons (**b**). concentration of polycyclic aromatic hydrocarbons (PAHs) (**c**). T0-ctr and cons. (treatments: control and consortium, time zero); T0-oil and oil+cons. (treatments: oil and oil+consortium, time zero); T10-consortium (consortium, day 10 of the experiment); T10-oil+consortium (oil+consortium, day 10 of the experiment); T10-oil (oil only, day 10 of the experiment).

The initial concentration of total petroleum hydrocarbons (TPH) in the oil-treated microcosms was 949.82 (±157.92) mg L^−1^. After 10 days, this concentration decreased to 693.76 (±35.02) mg L^−1^ and 258.86 (±23.22) mg L^−1^ in the T10-oil and T10-oil + consortium treatments, respectively (Fig. 1a). The decrease in the bacterially-treated systems was significantly higher that in the untreated ones (P < 0.05). Thus, ‘natural’ degradation, represented by the T10-oil treatment with no added consortium, removed 27.1% (±2.4) of the initial TPH load. In contrast, bioremediation removed 72.75% (±3.7%) of the initial load.

With respect to n-alkanes, the degradation rates were considerable, but these did not differ significantly (P > 0.05) between the T10-oil and T10-oil + consortium treatments (Fig. 1b). The initial concentration of n-alkanes was 189.91 (±85.78) mg/L, which was reduced to 55.42 (±4.78) mg/L and 40.76 (±4.83) mg/L after 10 days in the T10-oil and T10-oil + consortium treatments, respectively.

Lower concentrations of 38 polycyclic aromatic hydrocarbons (PAHs) were found in the T10-oil + consortium treatment compared to the T10-oil treatment (Fig. 1c). The initial concentration of PAHs was 64.45 (±8.43) mg L^−1^, which decreased to 55.67 (±15.80) and 28.20 (±9.45) mg L^−1^ after 10 days in the T10-oil and T10-oil + consortium treatments, respectively (Fig. 1c). Thus, ‘natural’ degradation, represented by the T10-oil treatment, was approximately 13.6% (±24.5%), whereas the degradation in the T10-oil + consortium treatment was 56.2% (±14.7%).

Among the PAHs, naphthalenes (naphthalene; 2-methylnaphthalene; 1-methylnaphthalene; C2 naphthalene), acenaphthene and fluorine were notable for their faster disappearance in the presence of the bacterial consortium; 100%, 75% and 65% of these compounds were degraded, respectively, in the T10-oil + consortium treatment. In contrast, in the T10-oil treatment, these values were 49, 35 and 15%. Expectedly, the phenanthrenes (phenanthrene; C1 phenanthrenes; C2 phenanthrenes; C3 phenanthrenes; C4 phenanthrenes) which are more recalcitrant hydrocarbons, were not degraded by the T10-oil + consortium nor by the T10-oil treatments (data not shown).

### Protection of M. harttii photochemical ability by the bacterial consortium

**Figure 2:**
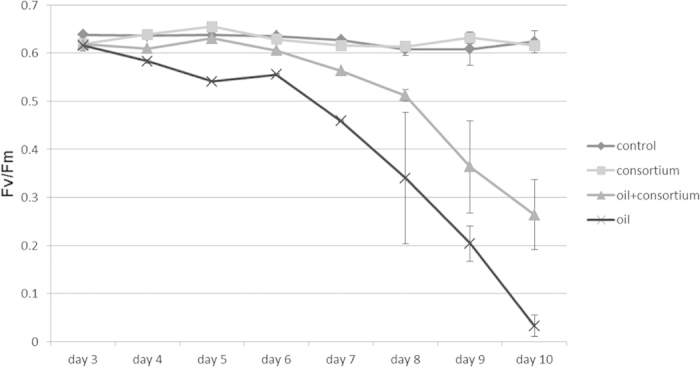
Dark-adapted F_v_/F_m_ measured in *M. harttii* using a diving-PAM chlorophyll fluorometer. Measurement performed at 17 h. Abbreviations: T0-control (control, time zero); T10-control (control, day 10 of the experiment); T10-consortium (consortium, day 10 of the experiment); T10-oil (oil, day 10 of the experiment); T10-oil+consortium (oil and consortium, day 10 of the experiment).

A significant difference in *F*_*v*_/*F*_*m*_ was observed between treatments (F = 46.080; df = 3; p < 0.001) and over time (F = 18.035; df = 7; p < 0.001). The effect of treatments was strongly influenced by the exposure time as evidenced by a significant interaction of these two factors (F = 5.703; df = 21; p < 0.001). The most pronounced time effect was observed in the exposed coral polyps to oil WSFs, which caused a progressive decrease in *F*_*v*_/*F*_*m*_ from the 4th day onwards (Fig. 2). This decrease became more pronounced on the 6th day of incubation, reaching values close to zero by the 10th day. In contrast, photochemical ability was clearly preserved in the systems with the bacterial consortium, which was not significantly different when compared to the control treatment. In fact, whereas the *F*_*v*_/*F*_*m*_ metric decreased by 95% after 10 days of exposure to oil in the systems without the bacterial consortium, which was significantly different when compared to other treatments (p < 0.0001). In the presence of the bacterial consortium the corresponding reduction was only 57%, however, still significantly different from control treatment (p < 0.001).

### Responses of biomarkers of coral health/activity

**Figure 3:**
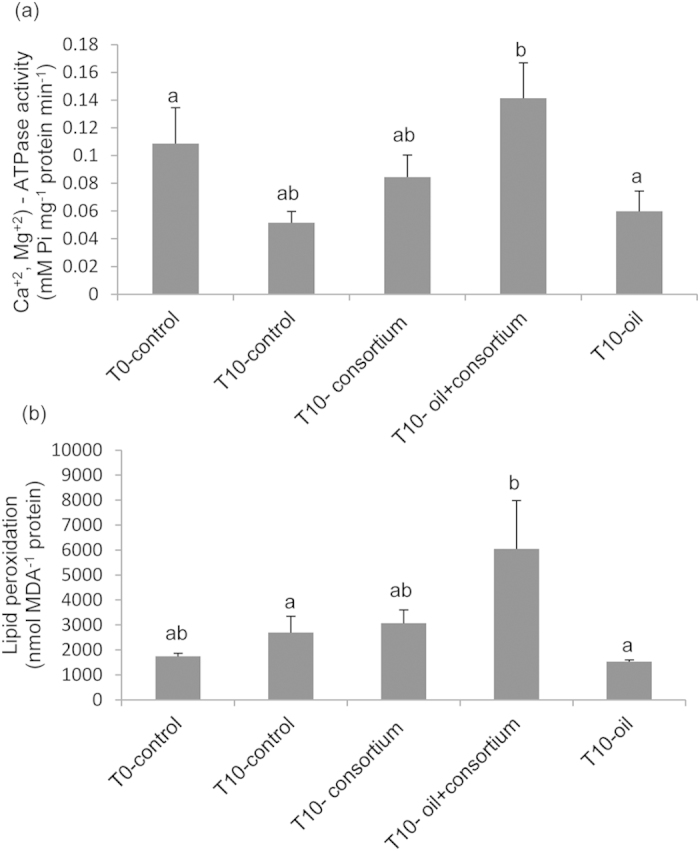
(Ca^2+^, Mg^2+^)-ATPase activity. (**a**). lipid peroxidation (**b**). Coral samples were subjected to oil exposure and bioremediation. Abbreviations: T0-control (control, time zero); T10-control (control, day 10 of the experiment); T10-consortium (consortium, day 10 of the experiment); T10-oil (oil, day 10 of the experiment); T10-oil + consortium (oil and consortium, day 10 of the experiment).

At day 10, (Ca^2+^, Mg^2+^)-ATPase activity showed a significant increase (p < 0.05) in the coral exposed to the oil + consortium treatment when compared to coral exposed only to oil (Fig. 3a). Lipid peroxidative damage (LPO) levels followed a similar trend. We observed significant differences (p < 0.05) between the T10-control and T10-oil + consortium treatments, as well as between the T10-oil and T10 oil + consortium treatments (Fig. 3b).

### Effects of oil on the coral microbiome

**Figure 4:**
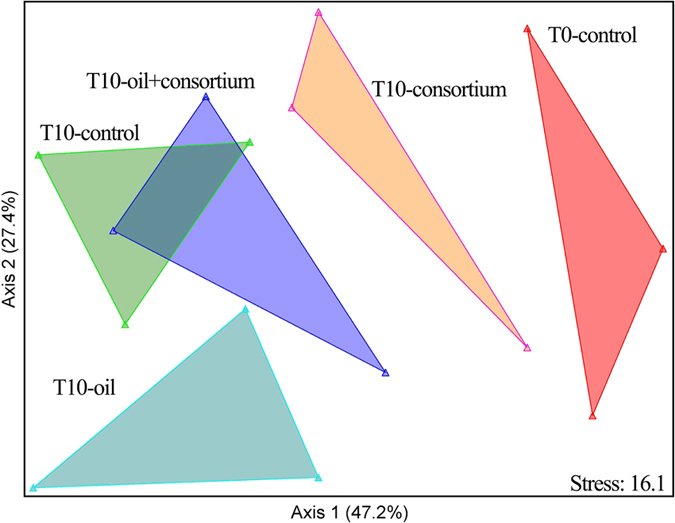
NMDS ordination of the partial 16S rRNA gene sequences of the bacteria associated with the coral *M. harttii.* Abbreviations: T10-control (control, day 10 of the experiment); T10-consortium (consortium, day 10 of the experiment); T10-oil (oil, day 10 of the experiment); T10-oil + consortium (oil and consortium, day 10 of the experiment).

The corals of the T10-control and T10-oil + consortium treatments were found to contain similar OTU clusters that were different from those of T10-oil (Fig. 4). This result was corroborated by PCR-DGGE analyses of the total bacterial communities and was consistent between replicates (Supplementary Fig. 1).

**Figure 5:**
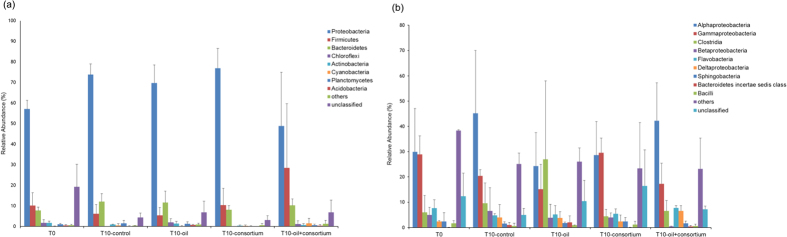
Relative abundances of partial 16S rRNA gene sequences of the bacteria associated with the coral *M. harttii* under different treatment conditions, as calculated using RDP-Classifier. Relative abundances of the most abundant phyla (**a**). relative abundances of the most abundant classes (**b**).

In total, 28 different bacterial phyla were associated with *M. harttii*. Irrespective of treatment, all of the coral-associated bacterial communities were dominated by Proteobacteria, with showed relative abundances of 50–70%. Across all treatments, the second-most abundant phylum was Firmicutes, and the third was Bacteroidetes (Fig. 5a). Alterations in the relative abundances of bacterial taxonomic groups associated with *M. harttii* became clear when different individual classes were analysed (Fig. 5b). Ten days after contamination with oil (T10-oil), there was a decrease of the class Alphaproteobacteria and an increase of the class Clostridia compared to the control treatment (T10-control). Remarkably, in the T10-oil + consortium treatment, these alterations were not seen.

**Figure 6:**
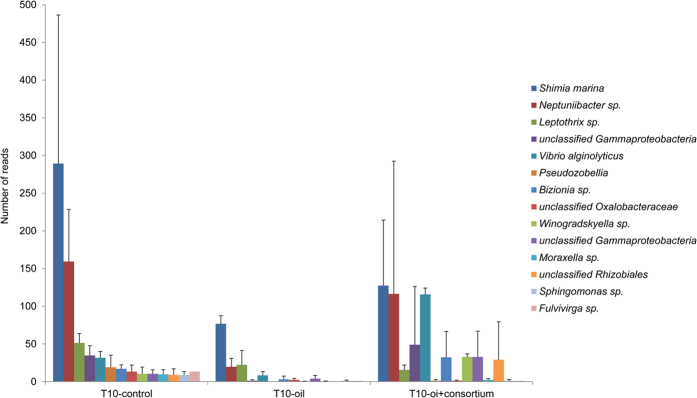
The 100 most abundant OTUs that significantly decreased (P < 0.05) from the T10-control treatment to the T10-oil treatment and that decreased or remained steady between the T10-control treatment and the T10-oil + consortium treatment.

We then identified the OTUs that decreased significantly (P < 0.05) in the T10-oil and T10-oil + consortium treatments when compared with the T10-control one. For this analysis, the 100 most abundant OTUs in the control were evaluated. Significant decreases in the relative abundances of 14 (/100) OTUs in the T10-oil treatment were found (Fig. 6). Only five of these also showed significant decreases in the T10-oil + consortium treatment. The 9 OTUs that did not decrease in the oil + consortium treatment, with one exception, did not match any consortium member. Thus, these were affiliated, at >97% similarity, with *Vibrio alginolyticus*, *Neptuniibacter sp*., *Shimia marina, Bizionia sp*., *Winogradskyella sp.*, *Fulvivirga sp.*, an unclassified member of the Rhizobiales and 2 unclassified Gammaproteobacteria.

Indeed, the OTU affiliated with *V. alginolyticus* that had decreased significantly in the T10-oil treatment increased significantly in the T10-oil + consortium, possibly due to the presence of *V. alginolyticus* (strain I9) in the consortium.

## Discussion

This study addressed the impact of an oil spill on the *M. harttii* holobiont in microcosms closely mimicking natural conditions. Oil WSF were used, as the WSFs presumably have the greatest impact on coral health. We then evaluated whether the impact of the oil WSF was modulated by an assembled bacterial consortium. As expected, oil WSFs impacted *M. harttii* health in varying (negative) ways, and they also affected the *M. harttii* microbiome. Remarkably, the bioremediation strategy, which was based on a single dosage of a consortium composed of 10 oleophilic morphotypes selected on a specific growth medium already improved *M. harttii* health. It also significantly accelerated the degradation of petroleum hydrocarbons.

The bacterial consortium generated in this study was assembled on the basis of 10 different, novel bacterial strains that had been grown on a WSF-containing medium. Some of the bacterial strains were affiliated with species that are known to be capable of degrading oil hydrocarbons^17^,^18^,^19^,^20^,^21^. Indeed, the TPH degradation efficiencies obtained with the bacterial consortium (66.6 ± 10.4%) were high as compared to those reported in other studies^22^,^23^,^24^. Not surprisingly, the n-alkanes hydrocarbon degradation rates were high even under natural degradation conditions. This result is consistent with those of Souza and colleagues^25^, who revealed that the natural degradation of alkanes in seawater reached 66% after 4 days.

PAH compounds, due to their chemical structures, are much more recalcitrant to degradation^26^,^27^. Our results revealed remarkably efficient PAH degradation by the applied bacterial consortium. In the T10-oil + consortium treatment, the degradation was approximately 43% greater than that of the T10-oil treatment. However, we ignore the effect of, for instance, differential volatilization of WSFs and/or of other factors such as adsorption and fungal degradation as other potentially confounding factors that affected the results. The PAH compounds that were most strongly degraded by the bacterial consortium were naphthalenes, acenaphthene and fluorine. During an oil spill at a coral reef containing many sensitive organisms, naphthalenes likely have the highest impact because of their toxicity and high water solubility (31 mg L^−1^)^28^.

The impact of oil WSFs on the health of *M. harttii* was assessed by determining the photosynthetic capacity of zooxanthellae. The maximum quantum yield of Photosystem II (*F*_*v*_/*F*_*m*_) showed a progressive decrease that was proportional to the exposure time to WSFs. However, the bacterial consortium was able to significantly reduce the negative effect of oil without significant side effect. Previous studies regarding the effects of WSFs from the Harriet oil field (Australia) on the photosynthetic capacity of the coral *Plesiastrea versipora* showed that a significant reduction in *F*_*v*_/*F*_*m*_ in the first 48 h occurred only in treatments with WSF concentrations above 12.5% (v/v)^29^. The lowered *F*_*v*_/*F*_*m*_ values suggest that the repair processes of damaged Photosystem II reaction centres were reduced, as also occurs with osmotic, heavy metal and nutrient limitation stresses^30^. Moreover, in colonies of *Acropora formosa* exposed to hydrocarbons originating from lubrification oils, extrusion of zooxanthellae was observed concomitantly with decreases in *F*_*v*_/*F*_*m*_^31^. Such extrusion might relate to a process of ‘removal of damaged parts’ by the coral, as post-disturbance recovery of *F*_*v*_/*F*_*m*_ was associated with the selective loss of damaged zooxanthellae (i.e., those with lower *F*_*v*_/*F*_*m*_)^32^.

We further inferred that, as a result of the exposure to oil WSFs, there was a significant increase (p < 0.05) in oxidative damage (lipid peroxidation) and (Ca^2+^, Mg^2+^)-ATPase activity after 10 days in the presence of the added bacterial consortium. It is possible that the consortium generated oxidative stress that spurred the activity of the related enzymes. As lipid peroxidation can modify membrane structures, this alteration may affect the function of membrane-bound enzymes^33^,^34^,^35^ as (Ca^2+^, Mg^2+^)-ATPase. Calcium and magnesium ion transport activity may have increased to compensate for a possible ionic imbalance due to oxidative stress-induced changes in coral membrane permeability.

The Ca^2+^-ATPase pump of animal cell plasma membranes maintains low internal concentrations of Ca^2+^, and calcium pump disruption in corals can lead to high intracellular concentrations of Ca^2+^, thus resulting in blebbing or ballooning out of the membrane and bleaching^35^,^36^. It is has also been hypothesised that Ca^2+^-ATPase has a primary role in the coral calcification process. This enzyme may transport Ca^2+^ into the calcification site while removing protons from it, thereby driving the calcification reaction towards the formation of CaCO_3_^37^. In turn, it has been proposed that Mg^2+^-ATPase enzyme activity is used to actively control the growth of different skeletal components^38^, which also makes this enzyme an important player in the process of coral calcification. Given all of these observations, we suggest that the Ca^2+^-ATPase pump would have to work harder to maintain low intracellular concentrations of Ca^2+^; simultaneously, in cells of the calicoblastic layer, more of the leaked Ca^2+^ would be transported out to the site of calcification. A similar mechanism has been proposed to explain the role of lipid peroxidation in the higher calcification rates observed during daylight in corals, as well as to explain the higher calcification rates found in hermatypic as compared to ahermatypic and deep-sea corals^39^. Therefore, an increase in the active transport of calcium/magnesium may minimise the impact of microbial activity on coral physiology during WSF degradation, thus benefiting the calcification process.

We surmised that *M. harttii* must tolerate lipid peroxidation during WSF degradation (i.e., it avoids membrane disruption), as it exhibited lower decreases in its maximal photosynthetic efficiencies in the oil + consortium treatment. The calcification process may benefit from this protective response, thus resulting in heightened calcification rates. Moreover, corals dispose of mechanisms that repair lipid peroxidation damage^35^,^40^. Revealingly, the impact of oil WSFs on the coral-associated bacterial community was minimised by the action of the added bacterial consortium. This protective effect was possibly related to the high efficiency of WSF degradation by the added bacterial consortium. In addition, probiotic (‘health-enhancing’) effects might have also occurred.

Among the OTUs that were ‘rescued’ by the bacterial consortium, we highlight those affiliated with *Shimia marina, Neptuniibacter sp.,* next to *Vibrio alginolyticus. S. marina* belongs to the clade Roseobacter, which is one of the most abundant groups in marine environments^41^. *Roseobacter spp*. are commonly known for their beneficial activity in marine organisms, as they show antagonistic activity against the pathogens *Vibrio anguillarum*, *V. splendidus* and a *Pseudoalteromonas sp.*^42^. The genus *Neptuniibacter* is a key bacterial inhabitant of the Brazilian corals *Mussismilia hispida, M. braziliensis* and *M. harttii*^43^. It is possible that this organism performs an important role in maintaining the health of these corals, and it may have been essential for maintaining the health of corals in the oil + consortium treatment.

Moreover, *Vibrio alginolyticus* and the species of the genera *Bacillus* and *Pseudomonas* that were utilised to build the consortium are among key probiotic bacteria that have been proposed as biological control agents in aquaculture^44^. For example, strain I4, affiliated with *Bacillus aryabhattai,* is resistant to ultraviolet radiation (UV) and can solubilise zinc^45^,^46^. Its potential Zn-solubilisation activity is important, as the concentration of dissolved zinc in seawater is normally low (0.1 nM)^47^, and zinc plays an important role in zooxanthella-driven photosynthesis and in the calcification of corals^48^,^49^. Members of the genus *Bacillus* have also been reported to enhance water quality and to promote the survival, growth and health of juvenile *Penaeus monodon*. This genus may also reduce the presence of pathogenic *Vibrio* species, which are commonly reported as pathogens of corals^50^.

The species *Vibrio alginolyticus* was sensitive to contamination by oil, but it recovered in the oil + consortium treatment, possibly due to its presence in the consortium itself. It has been recommended as a probiotic for different marine organisms^51^,^52^. This species also exhibited some protection against disease in marine organisms, such as against pathogens of the genus *Vibrio*^53^.

Our results show that using probiotic microorganisms to improve the health of corals under stress can foster coral health and survival. Thus, the strategy proposed in this work may promote the survival of a coral reef. In contrast to existing chemical dispersant-based strategies that potentially cause harm to the coral^9^, our approach sets the stage for improved, environmentally friendly strategies.

## Methods

This study was divided into 2 steps. The first step was the construction of bacterial consortia (using the coral *M. harttii* as a source habitat) that were able to degrade oil WSFs. The second step was to evaluate the impact of the oil WSFs and the potential of the consortia to improve coral survival. The experiment was conducted in seawater microcosms (tanks) containing *M. harttii*. We addressed four different parameters: (i) the potential for petroleum hydrocarbon degradation (and coral protection) by the bacterial consortium; (ii) the impact of oil WSFs on the chlorophyll fluorescence of the coral symbionts (zooxanthellae); (iii) the biological oxidative-stress and calcification responses to the treatments; and (iv) the impact of oil WSFs on the bacterial community associated with the coral.

### Ethics statement

Permission for sampling was obtained from the Brazilian Institute of Environment and Renewable Natural Resources (IBAMA)/Chico Mendes Institute for Biodiversity Conservation (ICMBio), permanent permission number 16942, and from SMMA/Porto Seguro, in accordance with the Instruction Normative n° 03/2014 of System Authorization and Information on Biodiversity (SISBIO). All experimental protocol were approved by the Brazilian National Council for Scientific and Technological Development.

### Bacterial consortium–sample collection, isolation and identification

The bacterial consortium used in this study was obtained from *M. harttii* colonies collected at the Recife de Fora, Porto Seguro, Bahia, Brazil (16°24′ S, 038°59′ W). After collection, 3 samples were maintained at 4 °C for 6 hours on the vessel until processing. In the laboratory, 5 g of coral was macerated in 0.85% sterile saline solution (45 ml) and then shaken with glass beads for 3 h. Subsamples (100 μl) of 10^−1^, 10^−2^ and 10^−3^ dilutions were then introduced into 20 ml of BH medium (Bushnell-Haas Sigma/USA) supplemented with 4 ml of oil WSF. The oil WSF, to the strain isolation phase, was obtained by shaking (at 180 rpm) 100 ml of marine fuel oil MF-380 in 300 ml of sterile distilled water for 48 h. After shaking, the water phase containing the oil WSF was separated from the oil phase using a separation funnel.

Using this new culture medium, the 10 different morphotypes with the fastest growth were selected. These were streaked for isolation.

From each strain, genomic DNA was extracted using the Wizard Genomic DNA Purification kit (Promega, USA). The 16S rRNA genes were PCR-amplified from the genomic DNA samples with the bacterial primers 27f (5′-AGA GTT TGA TCA TGG CTC AG-3′) and 1492r (5′-GTT TAC CTT GTT ACG ACT T-3′)^54^. The PCR was performed with 5 μl of 10X buffer, 2.0 mM MgCl_2_, 0.2 mM dNTPs, 5 mM of each primer, 2–4 ng of genomic DNA and 2.5 U Taq DNA polymerase (Promega, USA) in a final volume of 50 μl. The thermal cycling protocol was as follows: 94 °C for 4 min; 35 cycles of 94 °C for 1 min, 50 °C for 1 min, and 72 °C for 2.5 min; and a final extension cycle of 10 min at 72 °C.

The amplicons were purified using the GFX PCR DNA and Gel Band Purification kit (GE Healthcare). The amplicons were commercially sequenced (Macrogen Inc., Seoul, South Korea) using primers 27f (5′-AGA GTT TGA TCA TGG CTC AG-3′), 1492r (5′-GTT TAC CTT GTT ACG ACT T-3′), 532 (5′-CGT GCC AGC AGC CGC GGT AA-3′) and 907 (5′-CCG TCA ATT CMT TTG AGT TT-3′)^54^. The sequencing electropherograms were processed using the Ribosomal Database Project Sanger Pipeline (RDP; http://pyro.cme.msu.edu) to remove low-quality sequences. The sequences from each isolate were assembled into contigs using the program Bioedit 7.0.5.3^55^. The phylogenetic tree was constructed and edited using MEGA 5.0 and the Jukes-Cantor method^56^. Maximum-likelihood dendrograms were generated with bootstrap values of 1,000. The sequences were deposited in GenBank under accession numbers KR108381-KR108390.

### Experimental design

**Figure 7:**
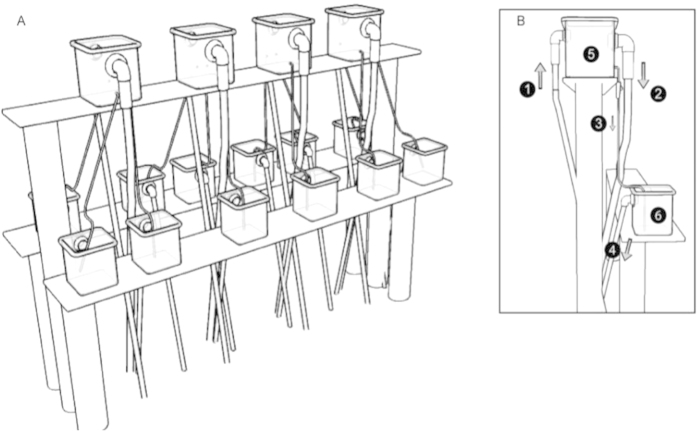
System for oil-contamination studies in coral. (**A**) system overview (the treatment tanks where the treatments were prepared are not shown in the figure); (**B**) detailed view of a replicate. 1, supply hose from the treatment tank (not shown) to the distribution tank; 2, return hose from the distribution tank to the treatment tank (flow controlled); 3, supply hose from the distribution tank to the aquarium for coral cultivation; 4, hose for discarding; 5, distribution tank; 6, aquarium for coral cultivation.

The experiment was conducted for 10 days at the Coral Vivo Research Base, Bahia, Brazil. The seawater used in the experiment was collected on a reef across the research base. The same water was used for all treatments and all treatments were at the same conditions of irradiance and temperature. The water temperature was around 27 °C, and the water parameters, from experience, fluctuated within narrow borders (pH 8.1; DO 2.85 mg/L; OM 2.82 mg C/L; salinity 36 ppm). The experimental set-up encompassed 1,000-L water tanks (master tanks) interconnected with 4-L (elevated) feeder tanks, to form a circulating loop. Water was pumped from the bottom of the tanks to avoid the floating non-soluble fraction of the oil. In these master tanks, all treatments (control, oil, consortium, oil + consortium; oil used MF-380, 1% (v/v) were applied. In the experiment, we used oil added to seawater, and simply let the WSFs affect the actual microcosms by the flux that was applied; this simulated, to the best of our abilities, a bulk seawater oil spill affecting coral. Treatments with oil (oil and oil + consortium) were assembled using the same stock solution of seawater plus oil 1% (v/v), previously prepared. It was performed to ensure the same concentration of oil to all treatments. The coral/seawater microcosms (triplicates per treatment) consisted of 2-L microcosm (aquarium) systems containing polyps of the scleractinian coral *M. harttii* each (collected from 3 different areas of the reef) that were fed from the 1000-L “master tanks”, by letting in 4% of the circulating seawater (flow rate 1 L/h; controlling oxygenation; Fig. 7b). Each microcosm contained 3 polyps of coral, where one polyp was used to measure chlorophyll fluorescence, one was used to assess the bacterial community and the other was kept as a reserve. The microcosms were, thus, under a continuous flux of differentially treated seawater from the “master tanks”. This implied three replicates per treatment.

### Detection of petroleum hydrocarbons

Sampling to evaluate the concentration of petroleum hydrocarbons was performed from each of the triplicate microcosms using individual sterile amber glass bottles with teflon cap. All samples were stored chilled to 4 °C for 24 h. To efficiently evaluate the degradation of petroleum hydrocarbons by the bacterial consortium, the concentration of total petroleum hydrocarbons (TPH), n-alkanes hydrocarbons and polycyclic aromatic hydrocarbons (PAH) at the beginning of the experiment (T0) and 10 days after contamination (T10) were evaluated. The protocol used for hydrocarbon extraction was based on the US EPA 3510 method.

The TPH, n-alkanes hydrocarbons and PAH fractions were obtained by liquid chromatography on silica/alumina (7 g of deactivated alumina at 2%, 10 g of deactivated silica at 5%, and 1 g of sodium sulphate in a glass column 30 cm in length and 1.3 cm in internal diameter). TPH detection was performed by gas chromatography with flame ionisation detection (GC/FID) according to the US EPA method 8015, modified for hydrocarbon analysis. The n-alkanes hydrocarbons were identified and quantified by the internal standardisation method, using as an internal standard n-C24d (at a concentration of 2.5 μg/ml), and the 38 evaluated PAHs were detected by gas chromatography/mass spectrometry using a modified version of the US EPA 8270D method.

### Chlorophyll fluorescence measurements

The impact of oil WSFs on the health of *M. harttii* was assessed by determining the photosynthetic capacity of the associated zooxanthellae. For this determination, changes in the *F*_*v*_/*F*_*m*_ ratio (obtained from dark-adapted samples) were used to indicate the stress level imposed on the coral holobiont. The photosynthetic efficiency of the zooxanthellae was assessed using pulse-amplitude-modulated (PAM) fluorometry as a proxy for coral holobiont health. We used a submersible diving-PAM system (Walz GmbH, Effeltrich, Germany) fitted with a red-emitting diode (LED, peak emission at 650 nm) and an 8-mm standard glass fibre-optic probe, which was positioned above the oral disk of polyps. After dark adaptation for 20 min, the initial fluorescence signal (*F*_*o*_) was detected under the modulated measuring light of the PAM (a weak pulsed light; <1μmol photons m^−2^ s^−1^), and the maximal fluorescence level (*F*_*m*_) was estimated using a short saturating pulse of actinic light. The variable fluorescence (*F*_*v*_) was calculated from *F*_*m*_–*F*_*o*_, and the maximum quantum efficiency of Photosystem II (PSII) photochemistry was obtained from the ratio *F*_*v*_/*F*_*m*_. Measurements were conducted daily, at 17:00, when was measured one polyp in each microcosm. The diving-PAM was configured as follows: Measuring Light Intensity (MI) = 6; Saturation Pulse Intensity (SI) = 8; Saturation Pulse Width (SW) = 0.8, Gain (G) = 1; and Damping (D) = 1. A Two-way ANOVA, followed by Tukey post-hoc test, was performed with Systat 13 (Systat Software Inc.) to assess the significance of differences in *F*_*v*_/*F*_*m*_ ratio among treatments over time (treatments and time as independent variables).

### Biochemical biomarkers

Biochemical biomarkers were analysed to quantify oxidative damage in the coral *M. harttii* (peroxidative damage to lipids, LPO) and to assess the activity of key enzymes (i.e., (Ca^+2^, Mg^+2^)-ATPase) in the coral calcification process. Sample preparation for biomarker analyses was performed as described by Downs^57^, with modifications. Briefly, the samples were ground in liquid nitrogen, and aliquots (150–200 mg) were sonicated (Sonaer Ultrasonics, Farmingdale, NY, USA) on ice using the specific homogenisation buffer (1:2 w/v) required for the analysis of each biomarker, as described below. The homogenised samples were centrifuged (13,000 g) at 4 °C for 10 min. The intermediary phase was collected and immediately used for biomarker analysis. The total protein content in the supernatant was determined using a commercial reagent kit based on the Bradford assay (Sigma-Aldrich, St. Louis, MO, USA).

The LPO measurement was performed using the fluorimetric method described by Oakes and van der Kraak^58^, which is based on 2-thiobarbituric acid-reactive substances (TBARS). This assay quantifies the peroxidative damage to lipids by the reaction between malondialdehyde (MDA), a product resulting from lipid peroxidation, and thiobarbituric acid (TBA). The resulting fluorescence (excitation, 515 nm; emission, 553 nm) was measured using a fluorometer (Victor 2, Perkin Elmer, Waltham, MA, USA). The data were normalised to the total protein content in the sample homogenates and expressed as nmol MDA mg^−1^ protein.

The enzyme activities of (Ca^2+^, Mg^2+^)-ATPase were measured using the method described by Vajreswari *et al.*^59^, with modifications. Sample homogenates were prepared using a buffer solution containing 100 mM Tris-HCl (pH 7.6), 500 mM sucrose, 1 mM DTT, and 1 mM PMSF. The reaction solution used for the analysis contained 80 mM NaCl, 5 mM MgCl_2_, 0.5 mM CaCl_2_, and 20 mM Tris-HCl (pH 7.6), and the reaction was incubated at 30 °C for 30 min. Inorganic phosphate (Pi) released by the enzyme in the reaction medium was measured using the Fosfato commercial reagent kit (Doles, Goiás, Brazil), which is based on the colorimetric method described by Fisk and Subbarow^60^. Measurements were performed at 630 nm using a microplate reader (ELx-800, Biotek, Winooski, VT, EUA). The data were normalised to the total protein content in the sample homogenates and expressed as mM Pi mg^–1^ protein min^−1^.

### DNA extraction

To assess the bacterial community associated with the coral *M. harttii*, 0.5 g of 1 polyp from each aquarium (3 aquariums per treatment) was macerated in a mortar in dry conditions using a pestle. Total community DNA extraction was performed using a ZR Soil Microbe DNA kit (Zymo Research, USA)^61^. The DNA concentration was evaluated using a Qubit fluorometer.

### Sequencing of the 16S rRNA gene

The 16S rRNA gene V4 variable region PCR primers 515/806^62^ were used in a single-step, 30-cycle PCR using the HotStarTaq Plus Master Mix kit (Qiagen, USA) under the following conditions: 94 °C for 3 minutes, followed by 28 cycles (5 cycles used on PCR products) of 94 °C for 30 seconds, 53 °C for 40 seconds and 72 °C for 1 minute, after which a final elongation step at 72 °C for 5 minutes was performed. Sequencing was performed at MR DNA (Shallowater, TX, USA) on an Ion Torrent PGM by following the manufacturer’s guidelines.

### Bioinformatics analysis

The raw sequences were processed using Mothur v.1.33^63^. To reduce the error in the retained data set, all sequences that failed to comply with any one of the following criteria were excluded: average quality lower than 25, length under 200 bases, the presence of ambiguities, more than 1 nucleotide mismatch to the primer and/or barcodes, or homopolymers longer than 8 nucleotides. The remaining high-quality sequences were then aligned using Mothur and the Silva reference database^64^, and chimaeras were detected with chimaera.uchime. The sequences were then taxonomically classified using the Greengenes reference database^65^ with a 50% confidence threshold, and all sequences not classified into the Bacteria root were discarded. The resulting alignments, which contained only high-quality sequences, provided input for constructing the distance matrix and for clustering the sequences into operational taxonomic units (OTUs).

Clusters were constructed with a 3% dissimilarity cutoff and normalised to the number of sequences (selected randomly by Mothur). These clusters served as OTUs for generating predictive rarefaction models and for determining non-parametric species-richness estimators, such as abundance-based coverage estimators (ACE), Chao1^66^, and the Shannon diversity index^67^. Mothur software was used to screen for significant differences in the relative abundances of the most abundant OTUs, and a consensus taxonomic assignment for each OTU was performed.

Lastly, a matrix of OTU distributions among all of the samples was constructed. The matrix was ordinated using NMS^68^,^69^ with the Sørensen distance^70^ and a random initial configuration. The significance of the matrix structure was assessed using a Monte Carlo test.

## Additional Information

**How to cite this article**: Santos, H. F. *et al.* Impact of oil spills on coral reefs can be reduced by bioremediation using probiotic microbiota. *Sci. Rep.* **5**, 18268; doi: 10.1038/srep18268 (2015).

## Supplementary Material

Supplementary Information
