# Supplementary material for: Impact of oil spills on coral reefs can be reduced by bioremediation using probiotic microbiota
Source: Sci Rep. 2015 Dec 14;5:18268. doi: 10.1038/srep18268 (PMC4677405; doi:10.1038/srep18268)
Supplement: Supplementary Information [file srep18268-s1.pdf]

Impact of oil spills on coral reefs can be reduced by bioremediation using probiotic microbiota

Henrique Fragoso dos Santos, Gustavo Duarte, Caio Tavora Coelho da Costa Rachid, Ricardo Chaloub, Emiliano Nicolas Calderon, Laura Fernandes de Barros Marangoni, Adalto Bianchini, Adriana Haddad Nudi, Flavia Lima do Carmo, Jan Dirk van Elsas, Alexandre Soares Rosado, Clóvis Barreira e Castro, Raquel Silva Peixoto

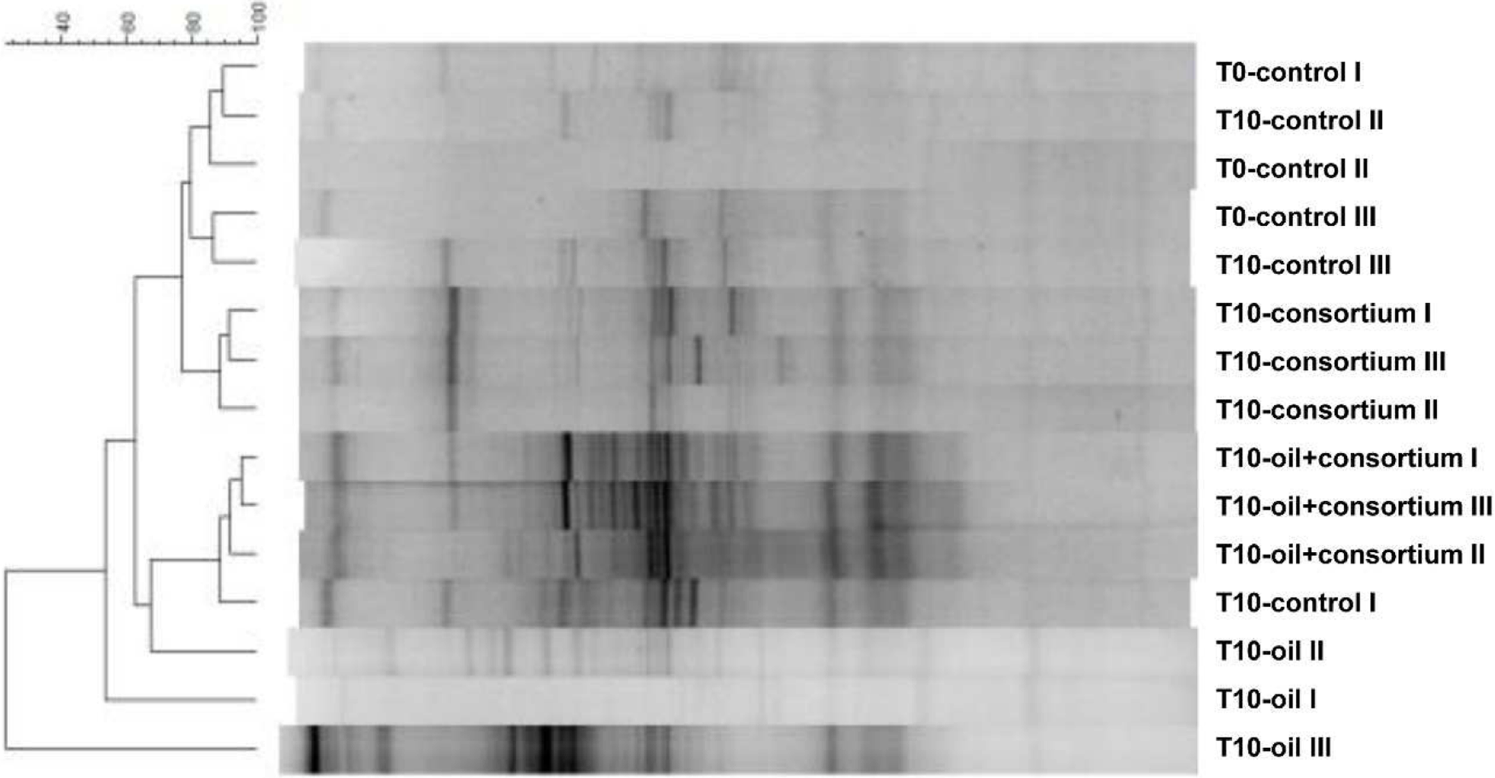

Figure S1. Dendrogram of PCR/DGGE profiles for 16S rRNA gene created using the UPGMA method based using Bionumerics Software. T0-control (control, time zero); T10-control (control, day 10 of the experiment); T10-consortium (consortium, day 10 of the experiment); T10-oil (oil, day 10 of the experiment); T10-oil+consortium (oil and consortium, day 10 of the experiment).
